# Joint Hypermobility Syndrome and Dysautonomia: Expanding Spectrum of Disease Presentation and Manifestation

**Published:** 2010-04-01

**Authors:** Shomu Bohora

**Affiliations:** Baroda Heart Institute and Research Centre, Vadodara, India

**Keywords:** Dysautonomia, Joint Hypermobility Syndrome

"Somehow our devils are never quite what we expect when we meet them face to face".  Nelson DeMille.

"The mind can cook up very subtle syndromes to throw at our bodies".  Astrid Alauda.

The varying clinical presentations of not so well recognized diseases, which continue to haunt patients is well described in the above quotes. Clinicians may not recognize associated subtle early presentations of the disease, till the disease has fully manifested itself. Of all the disorders of human body, autonomic syndromes are still the least understood. Presentation and association of autonomic diseases with other disorders may be so varied and subtle that clinicians may fail to recognize autonomic disturbance as an individual clinical entity within the disease and therefore appropriate management still remains inadequate. Similar association between joint hypermobility disorder, in which postural orthostatic tachycardia syndrome and dysautonomia is increasingly being recognized as an associated clinical entity, has been further elaborated.

Postural orthostatic tachycardia syndrome (POTS) is a form of dysautonomia affecting predominantly young females, consisting of a constellation of symptoms with symptomatic orthostatic intolerance and by definition, a change from supine to an upright position causes an increase in heart rate of more than 30 beats per minute or to a heart rate greater than 120 beats per minute within 10 minutes of upright position [1-3]. Tachycardia is often accompanied by a mild decrease in blood pressure as well. Sympathetic overactivity causing tachycardia, mild hypotension, autonomic dysfunction, brain and other organ hypoperfusion may cause a range of symptoms. Symptoms may range from mild and occasional complaints to severely incapacitating disease and are commonly misdiagnosed as having chronic anxiety or panic disorder or chronic fatigue syndrome [3,4]. POTS is often accompanied by vasovagal syncope [2-4].

Primary POTS is related to partial dysautonomia and hyperadrenergic state [2]. The cause of primary POTS is not fully known. Reduced venous return during standing due to venous pooling or denervation causing low plasma volume may be responsible for a hyperadrenergic state resulting as the body attempts to compensate for these abnormalities. Family history of orthostatic intolerance suggests a genetic predisposition [5]. Adrenergic receptor dysfunction [6] with alpha 1 and beta receptor supersensitivity, hyperdopaminergic state [7] and high free plasma norepinephrine levels have all been described with POTS. Precipitating physical or mental stress can trigger symptoms and disease which may thereafter persist for long [2,3].

Secondary POTS is due to peripheral autonomic denervation. Commonly associated conditions are diabetes mellitus, amyloidosis, sarcoidosis, alcoholism, lupus, heavy metal intoxication, Sjogren's syndrome and post-chemotherapy. Pure dysautonomic syndromes due to sympathetic under-activity may have POTS as a presenting feature. POTS has also been associated with musculoskeletal disorders like Ehlers-Danlos syndrome, Marfans syndrome, fibromyalgia and joint hypermobility syndrome.

Joint Hypermobility syndrome (JHS) was first described by Kirk et al in 1967 to describe a disorder of generalized joint hypermobility and musculoskeletal pain [8]. The term benign has been used in order to distinguish this symptomatic, but not life threatening, disorder from diseases such as Marfans and Ehlers-Danlos syndrome.  The prevalence of joint hypermobility varies from 10-30% and decreases with age [9-12]. Generalized hypermobility is thought to be a disorder of collagen that contributes to a loss of tensile strength and consequent increased fragility of involved tissues. As far as etiology of JHS is concerned, genetic predisposition (polygenic trait) is the most likely cause [13,14]. Also mutations in the TNXB gene have been associated with hypermobility [15,16]. In addition to the skin and musculoskeletal manifestations, mitral valve prolapse [17,18] and autonomic symptoms [19-22] are commonly seen. Surprisingly a large proportion of patients have significant neuromuscular and motor development problems in association with JHS [23].

In the current issue of this journal Kanjwal et al [24] have described clinical profile of patients having POTS with and without JHS.  In this retrospective study, they showed that patients with JHS and POTS appear to become symptomatic much earlier and have significantly higher incidence of syncope and migraine than the non JHS counterparts. Increase in joint laxity causing increased venous pooling with secondary hyperadrenergic state or receptor dysregulation predisposing to autonomic dysregulation has been postulated to be a cause of the increased association of POTS and syncope with JHS and abnormal vascular reactivity within the cerebral vasculature for migraine association with JHS [25-29]. There are no studies to suggest primary affection of the nervous system in JHS presently, though nearly 70% of patients of JHS may suffer from some form of dysautonomia related symptoms [2]. Based on the present study by Kanjwal et al [24], it may be appropriate that all young patients who present with symptoms of migraine or syncope, who have symptoms and orthostatic stress suggesting POTS should also be screened for hypermobility of joints and for JHS. In future dysautonomia related symptoms possibly would be considered for diagnosis of JHS in addition to the present revised Brighton diagnostic criteria for JHS which incorporates only musculoskeletal criteria for diagnosis of the condition [30].

There still remain some unanswered, but important, questions. How exactly are JHS and POTS associated? Why do both POTS and JHS have female preponderance? Is it related to genetic, hormonal or environmental modification of the nervous system along with differences in musculoskeletal structure in patients that predispose to the above or is there a single cause of all these contiguous syndromes which we are yet to discover? Based on what was remarkably said by Charles Dickens "Oh the nerves, the nerves; the mysteries of this machine called man!  Oh the little that unhinges it, poor creatures that we are!" we possibly would continue to recognize new syndromes of dysautonomia and its associations with various clinical entities out of this machine called man.
